# The Acceptability of Acupuncture for Low Back Pain: A Qualitative Study of Patient’s Experiences Nested within a Randomised Controlled Trial

**DOI:** 10.1371/journal.pone.0056806

**Published:** 2013-02-21

**Authors:** Ann Hopton, Kate Thomas, Hugh MacPherson

**Affiliations:** 1 Department of Health Sciences, University of York, York, North Yorkshire, United Kingdom; 2 Health Services Research Unit, University of Sheffield, Sheffield, South Yorkshire, United Kingdom; California Pacific Medical Center Research Institute, United States of America

## Abstract

**Introduction:**

The National Institute for Health and Clinical Excellence guidelines recommend acupuncture as a clinically effective treatment for chronic back pain. However, there is insufficient knowledge of what factors contribute to patients’ positive and negative experiences of acupuncture, and how those factors interact in terms of the acceptability of treatment. This study used patient interviews following acupuncture treatment for back pain to identify, understand and describe the elements that contribute or detract from acceptability of treatment.

**Methods:**

The study used semi-structured interviews. Twelve patients were interviewed using an interview schedule as a sub-study nested within a randomised controlled trial of acupuncture for chronic back pain. The interviews were analysed using thematic analysis.

**Results and Discussion:**

Three over-arching themes emerged from the analysis. The first entitled facilitators of acceptability contained five subthemes; experience of pain relief, improvements in physical activity, relaxation, psychological benefit, reduced reliance on medication. The second over-arching theme identified barriers to acceptability, which included needle-related discomfort and temporary worsening of symptoms, pressure to continue treatment and financial cost. The third over-arching theme comprised mediators of acceptability, which included pre-treatment mediators such as expectation and previous experience, and treatment-related mediators of time, therapeutic alliance, lifestyle advice and the patient’s active involvement in recovery. These themes inform our understanding of the acceptability of acupuncture to patients with low back pain.

**Conclusion:**

The acceptability of acupuncture treatment for low back pain is complex and multifaceted. The therapeutic relationship between the practitioner and patient emerged as a strong driver for acceptability, and as a useful vehicle to develop the patients’ self-efficacy in pain management in the longer term. Unpleasant treatment related effects do not necessarily detract from patients’ overall perception of acceptability.

## Introduction

Chronic low back pain is a problem in the UK. [1] Pain-related anxieties and fearful avoidance of movement are influential psychological factors from pain inception to its chronic stage. [2]; [3] Concerns about back pain raised through misinformation, learned pain, or distorted significance of the pain are associated with increased disability. [4] Tension between General Practitioners’ recommendation to stay active, versus the patients’ expectation of prescribed rest can lead to discord in the doctor-patient relationship. [5] Constraints on time and resources have led patients and doctors to feelings of frustration in the management of back pain in primary care [6]; [7].

The results of the York Acupuncture for Back Pain trial(YACBAC) [8] showed that a short course of acupuncture compared to usual care for chronic low back pain conferred a clinically significant reduction in low back pain for minor extra cost to the NHS. [9] Within the YACBAC trial, 241 participants, aged 18–65 with a history of non-specific low back pain for a period of 4 to 52 weeks were recruited by general practitioners at GP primary care practices in York. Participants were randomly assigned to receive a course of up to 10 acupuncture sessions over three months as an adjunct to usual care, or to receive usual care alone. Six acupuncturists with a minimum of three years’ experience and registered with the British Acupuncture Council delivered up to ten treatments, usually weekly, tailored to individual patients’ needs. Additional care such as brief massage and acupuncture-specific advice was provided if considered appropriate by the acupuncturists [10].

Evidence from a trialists’ collaboration which conducted a meta-analysis of nearly 18,000 cases of acupuncture for chronic pain conditions including non-specific back pain indicated that acupuncture for low back pain is more than a placebo, although the difference between true acupuncture and sham acupuncture is relatively modest. [11] This study suggested that factors in addition to the specific effect of needling are important contributors to therapeutic effects. Based on the growing evidence of clinical effectiveness and cost effectiveness, the National Institute for Health and Clinical Excellence guidelines recommend acupuncture as a referral option for patients with low back pain [12].

According to the MRC guidelines for the evaluation of complex interventions, an important part of an evaluation process is an exploration of the way in which the intervention under study is implemented, because it can provide valuable insight into why an intervention fails or has unexpected consequences, or why a successful intervention works and how it can be optimised. [13] Given the combination of physical, psychological and social components associated with chronic back pain, it remains unclear as to what additional factors contribute to patients’ positive and negative experiences of acupuncture and how these factors interact to influence the patients’ perception of the treatment. Understanding why a treatment is acceptable to some patients and not others is important because acceptability may influence the patients’ rating of clinical benefit. Quantitative data from the YACBAC trial shows on average that there is a reduction in the intensity of back pain [8], but we know little about the factors at an individual level that might influence a patients’ recovery and their subsequent decision to try acupuncture again. These qualitative data will help clinicians to be better informed about who might benefit from referral to acupuncture.

To extend our understanding of acceptability, we have used qualitative methods, based on in-depth interviews with patients who received acupuncture for chronic low back pain nested within a randomised controlled trial. Our aim has been to capture patients’ reports on their thoughts, attitudes and experiences of treatment and to understand and describe the elements that they ascribe to the acceptability, or not, of acupuncture.

## Methods

### Setting and Ethics Statement

This research was a qualitative sub-study nested within the York Acupuncture for Back Pain Trial (YACBAC)(ISRCTN80764175) [14] funded by the Health Technology Assessment programme. The trial was conducted collaboratively by researchers at the University of Sheffield and the Foundation for Research into Traditional Chinese Medicine in York, and approved by York’s NHS Local Research Ethics Committee. Written consent was obtained from all participants using a consent document and procedure approved by the ethics committee.

In a postal questionnaire at three months after randomisation, 133 participants reported on treatment effects of acupuncture. Of those, twelve interview participants were drawn as a purposive sample, designed to include a range of known patient characteristics: age and gender, previous experience of acupuncture treatment, and both good and less favourable treatment outcomes. All 12 participants approached agreed to participate in the study and consented to a one to one, face-to-face, audio-recorded, semi-structured interview in their own home, and were considered suitable to represent the diversity of known patient characteristics.

Lucy Thorpe, a research associate experienced in investigating acupuncture for back pain and depression and trained in conducting qualitative research recruited and interviewed all 12 participants, and Mike Fitter, a research consultant provided additional supervision.

The interview opened with an introduction designed to draw out the participants’ account of treatment received as part of the trial, followed by prompts from a prepared topic guide (Appendix S1) to elicit the participants’ expectations of treatment before receiving acupuncture, therapeutic aspects of the consultation and their experiences of the treatment received. Interviews typically lasted for approximately 30–60 minutes; audiotaped recordings were transcribed verbatim and checked for accuracy. The interviews were conducted according to the topic guide and provided rich detail without further need to re-interview the selected sample. Iterative questioning was used within the interview to establish credibility and trustworthiness of the interviewees. Statements regarding the reduction in back pain experienced, the degree of function regained, and aspects of satisfaction with the time, attention, information and explanations given by the acupuncturist about treatment were crosschecked with quantitative data recorded as part of the trial.

### Analytical Methods

An inductive thematic analysis following the methods of Braun & Clarke 2006 [15] was used to search across the dataset of the 12 transcribed interviews, to organise and describe the data in detail, and to actively identify, analyze and report over-arching themes and subthemes. Thematic analysis was selected as a flexible research method with the potential to allow the data to speak for itself and to provide a rich and detailed account of the data. The data was analysed initially by AH by reading each transcript several times to become immersed in the data, and identifying interesting features within individual data sets that might form the foundation of repeated patterns. All data sets were coded manually by annotating notes within the text, and using colour coding to highlight potential patterns. The inductive codes developed from the dataset captured and summarised the participants’ experiences. As codes were identified, recorded and organised on an Excel spread sheet, sections of text that demonstrated that code were then added and collated. Coding progressed by moving back and forth across the data set in an iterative process where comparisons were made between codes and phrases. Those with similar context or concepts were grouped together. Identified codes were then matched to data extracts that demonstrated the code and collated together by copying extracts from individual transcripts and inserting them into an Excel spread sheet. This coding was performed sequentially on individual transcripts without software, working systematically throughout the entire dataset. This process was conducted within each interview and across interviews resulting in a codebook of 43 codes and 21 subthemes. Data saturation occurred early with all the codes appearing within the first six interviews. Ongoing analysis refined the specific content of each theme, and the position of codes and themes on the thematic map.

**Figure 1:**
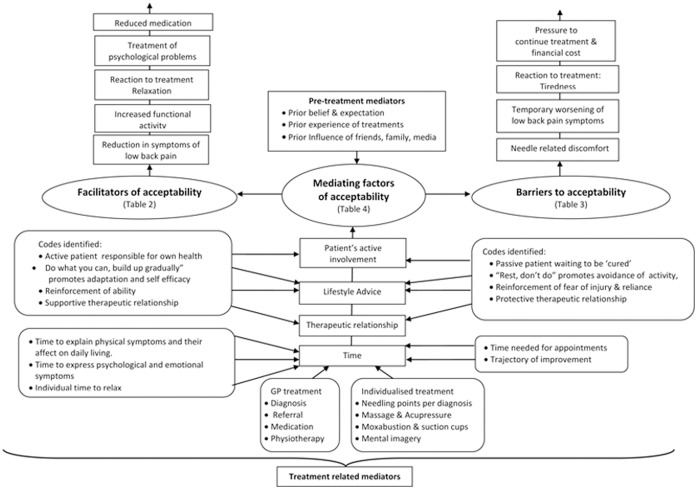
Initial thematic map showing three over-arching themes and related subthemes.

Three over-arching themes were identified and developed from the codes, Figure 1.

The data was checked independently by co-author HM, and the coding and identification of subthemes were discussed and developed collaboratively between AH and HM throughout. The subthemes are illustrated with quotes that embody the participants’ experiences embedded within the analytical narrative, as suggested by Braun and Clarke (2006). Additional quotes that support each subtheme within the over-arching themes are set out in Tables 1, 2, 3. To maintain anonymity each participant was numbered. Coding and extractions were checked to verify that the patient’s experiences were reflected and summarised accurately. The consolidated criteria for reporting qualitative studies (COREQ): 32-item checklist is available as (Appendix S2).

**Table 1:** Over-arching theme 1: Benefits of acupuncture.

*Reduction of symptoms of low back pain*
I am not going though as much of the aches and pains that I was. It has… it’s done some good. I still have the odd twinge and it was only the other day I felt like a tug but it seemed to stop quite quickly….I’d probably say I felt about 70% better. I’ve not felt 100% better. Before the acupuncture my back felt really weak, and since the acupuncture its felt stronger.(P2)
I’ve had no feeling at all down my calf and my ankle and my instep, and she’s even got rid of that….As each week goes by it gets better and there are some days when I just don’t feel it. I can forget about it now which is marvellous.(P7)
For me it was quite immediate…it was almost immediately I felt benefits. I felt that my foot had become alienated from my body because of the surgery… It was like a strange feeling that I had this foot but it was just like a lump, I didn’t have a bodily association with it… and then I did feel that because of the treatment the warmth and everything else and the way she sort of channelled everything I did feel that I had actually had again an association with my foot… its like she brought it back to me…(P9)
For the first couple of weeks I didn’t feel any difference and long term I did, as I say in the beginning, nothing,. Then it did start after a month then it started getting better and better every time I went, it was easier and easier..I didn’t seem to get sciatica as often… I must be honest when I bend over it still hurts, it still hurts when I wash my hair in the shower and I bend forward, … My pain is so much less than ever before over these years, so its been really great. I had a problem with my right shoulder as well. The muscle right inside your bone, that was really sore and I could hardly move, lift my arm and when It told the ladies about it at acupuncture, they were happy to work on it for me, … now I can lift my shoulder (P12)
*Improved physical activity*
I hadn’t walked for 2–3 months, you know pain does that to anybody I am sure…but I would have no qualms of setting out to do, well… ten mile walks., you know, with a 20 pound ruck sack on your back, so I guess I was back to normal… I still wash the car… bending down, and cleaning that will give me problems but its not giving me problems at the minute.(P1)
I’m much more mobile, I’m standing better, I can tolerate standing and sitting for slightly longer periods of time, and I can walk further. I’m sleeping better. I’m gradually doing a bit more.(P5)
The main thing was going back to work, the other thing was doing household chores. When I had my bad back I was trying to do the ironing, but I was doing like a shirt at a time and then lying down for five minutes and then doing another shirt…. It was horrendous trying to do anything… trying to do anything below waist level was virtually impossible. And I mean now, now I can do anything.(P7)
I think I’ve been able to focus more on what I can do, and not worry about that I can’t do… now I swim more because I can do that and I enjoy it and its good for you(P9)
*Relaxation*
You can generally feel something happening, its quite strange because you feel all tingly, you feel a bit warm, you feel tired… it does help relax you…The best part of it is just laying out and letting it be done to you.(P3)
I felt more relaxed in my back… that sort of feeling of well being and positive thoughts. That can change you physically at least for a while. I did actually find it relaxing lying on that couch thing with the needles in my back… conked out for a while… I’m more relaxed because I’m sleeping better as well.(P4)
I did feel extremely relaxed when I had treatment…she did actually give me a lot of energy… She did seem to channel quite a lot of energy and I felt that I had… after treatment I felt quite revitalized. It was really positive for me. I used to feel the next day that I had a lot, like a wham of energy and that she’d channelled through me, so it was really good.(P9)
I fell asleep; it was so relaxing, brilliant… I just used to lie there and relax and think oh I’ve got some freedom, some time to myself and I loved that…, it helps me clear my mind it gets me out, everything into one and of course its painless and its relaxing and it works.(P12)
*Psychological benefit*
Personally it did more for me head than it actually did for me back. you get a load of stress in your head and your body doesn’t work properly, you know that stress is transmitted into your posture and everything else. Some weeks she’d say right we’ll just completely forget about your back and just treat you for your emotional problems… (the acupuncturist) influenced that in making me feel better, giving me the ability to feel more positive about myself through going to see her. (P3)
Oh my God, my life is just going, where’s it going, suddenly days go into months and you just become aware of all sorts of things, time a, age, and everything. I haven’t been able to work… I’m getting worried about how I will ever get another job it’s a nightmare. I’m getting sick pay, it’s just above the line to be able to claim for income support, I’m building up enormous debt, I can’t pay the mortgage, I can’t pay it……. what can I do? when I am able I’ll man a reception, anything really, collating stuff, anything that’s within my ability… I feel I’ve been treated incredibly well. I feel I’ve been listened to… I felt supported, I felt listened to really. (P5)

**Table 2:** Over-arching theme 2: Barriers to acceptability.

*Needle-related discomfort*
One of the things about acupuncture is when you first start, you go the first one or two times and come out feeling slightly sore, not so much sore from the needles but just slightly worse in general. There seems to be a bit of an adverse effect and this has always happened. But as the success builds up then of course that doesn’t happen… it’s a dull sore ache but this was only for the first one or two times. My very last session… she really hit the bad spots with the needles and I asked her to take the needle out. It’s the first time in all my acupuncture that I’ve had real pain not needle pain, you know, the needle going in sharp, but the actual spot being hurt …(P1)
I got a twitching finger for four days, it was on the pressure point here, she’d hit a nerve. I had a very bad week with everything on the left hand side of my spine was agony every needle… she could scrape me off the ceiling with pain it was like a sword and I said you’re going to have to take it out and so she took it out and I could still feel the pain. (P6)
You’d be lying there waiting for it and it would be like an electric shock when she eventually got there, and my leg would react sort of jerk and it was Hush. Most sessions were quite painful, the actual insertion of the needles.(P7)
I had some needles in my hand for some reason… she had to take them out because my fingers just were numb, I got pins and needles so she said I’ll take them straight out. So she’d obviously hit a point where, probably a nerve or something. (P8)
*Temporary worsening of symptoms*
Sometimes when I got back I would feel, you know, a bit worse for wear. … one or two occasions it felt worse actually, but I’m not sure how much that’s then what I have gone on to do…. generally it only lasted a day and a night… I might not sleep so well that night, occasionally its gone on till I’ve had the next acupuncture, or almost… it’s just been wearing off when I’ve gone back for more torture.(P5)
The treatment did knock me about a bit to begin with, you know I felt worse to be honest. I’d got aches and pains all over my body and I was really, really, stiff.. I’d start to feel it within a couple of hours or so and then you start to feel all achey and thinking ‘oh golly’… is this really working, I feel worse now than I did before I went. (P8)
Five or six into the sessions when she nearly killed me… It just started really aching. Ached and ached and I got no sleep that night… It was really very, very, bad and it lasted the following day and the day after…it was just so severe, all the burning came back, I felt shattered the following day, no sleep (P10)
I had all that treatment yesterday and it hasn’t done any good for me, in fact I’m worse… I haven’t done anything since yesterday to make it worse… So why should I be so bad this morning? I mean it could be a positive thing I suppose.(P11)
*Tiredness*
I’m always glad I’ve been, and I always feel better after it, although it does tend to tire you out, but that’s ok, that doesn’t matter, you know it means you feel relaxed, very relaxed. I’d yawn a lot, yawn like the devil all the way home. I did it for the first three treatments. You know you feel a bit spaced out, you feel warm and a bit tingly/I had this tremendous yawning and desire to lay down, you know, which I do, I’d come back and lay down or just relax.(P3)
The first 2 or 3 sessions she said I’d be tired but I was literally yawning at the bus stop. It lasted for a couple of hours in the morning I think, Yeah, I found myself incredibly tired, Yawning away. Suddenly it seemed to come on, it was incredible. It didn’t happen every one, but I noticed it the first 2 or 3, you know I don’t normally stand yawning in the middle of the day (P4)
I used to feel very tired… It was just a progression maybe of just your body saying you know you needed time to heal, and I would just go with it and not want anything else from myself, just have the treatment and set that time aside just to go through.”(P9)
*Pressure to continue*
She talked about, well look, ten is not really enough, I think we should consider more…(P1)
I don’t think it is doing me any good you know, and yet I keep going and paying them money.. their care of you and their courtesy is you know second to none they are so nice, and the privacy and everything… that’s very special, and you don’t get that from your GP do you?(P11)
*Financial cost*
I felt she was trying to sell me things which I don’t think should have been part of the trial… I felt if I hadn’t thought about it, it could have been a bit of pressure on people… there is the potential for not so much being ripped off, but being misused. (P1)
I’d always longed to try acupuncture but couldn’t afford it because they won’t put it on the NHS….If my back plays up I will go for a session of acupuncture and I will get rid of it instead of moaning about it…as long as I can afford it. (P6)
She suggested some tablets so I said well I’ll give them a try, so I bought them off her and I got home and they were twelve months out of date, so I had to ring her up and say are they going to do me any harm? and she says well the strength will have gone out of them, so we left it alone and the problem cleared up on its own eventually. (P8)

**Table 3:** Over-arching theme 3; Mediating factors.

Pre-treatment mediators
*Expectation*
I know it works, … I am expecting a very high success rate from this trial.(P1)
It was something I had experienced before so I had no fear of it…I thought it will help.(P3)
My friends sister and her partner are both acupuncturists and they gave me some acupuncture and particularly (name) just literally used about four needles ad it helped tremendously, almost immediately. So therefore, I felt this was going to help.(P4)
I’m sure it (acupuncture) still does work for people. I’m sure that another form of acupuncture for me you know would work(P5)
I had to believe you see, you’ve got to believe in it, you’ve got to persevere…How on earth do you expect to get better if you don’t believe in it…I expected it to work more quickly.(P7)
I didn’t really know what I was hoping for I just wanted to get rid of this pain…I know of an instance second-hand where someone else has had treatment and it hasn’t done anything for them. (P8)
Prior I was always positive about going and my expectations were unknown because I didn’t know what to expect…I welcomed it really to give me the opportunity to try something that was away from trying drugs(p9)
I was a bit doubtful whether this would work… but quite willing to try it because I was in such a lot of pain…. (P11)
I was very excited, because anything to help my back problem I was prepared to try, anything…nothing else has helped… I couldn’t expect a complete cure for it (P12)
Treatment related mediators
*Time*
One of the interesting things is that they were 45 minute sessions and I had the needles in for say 35 minutes. Compared with when I am having it for my knee, it is no more than 10 minutes…. I know with Doc X if he carries on the treatment if I have any more back problems he will just do the 10 minute sessions.(P1)
I wasn’t benefiting (from acupuncture) and I found it interfered. If I was benefitting then I would have taken time aside, but I had started getting busy with work.(P4)
Generally speaking the impression you get at the doctors’ is that you’re one of hundreds of thousands, they want to get you in and out as quickly as possible. It’s not their fault that they haven’t got time.(P7)
She made me feel that when I went it was my time, that she was totally focussed on what she was doing for me and you know the continuity of the treatment… there was an hour set aside for me, instead of going to the Drs surgery and feeling that you know you had to remember everything you wanted to say, instead of doing that you were going to somebody you knew, you know you’d gone through the back ground they know what they were looking for, they knew what they were going to work on, and when you went they were prepared for you to continue treatment.(P9)
Blow the GP, because you wait to get in to see your GP, you can be waiting months and months to go for an appointment elsewhere, whereas all you’ve got to do is phone and say can I see so- an-so. (P10)
Part of it is slightly that Acupuncturist is busy going from room to room. There is little problem about that I think that you know that she is not with you all the time and she’s moving off to somebody else and then coming back and so she must be distracted, it’s quite difficult when she knows people are waiting for her, so she’s trying to give you the time, and yet she, so it’s the same system as in the Health Service, in the one sense in that yes there are always time constraints.(P11)
*The therapeutic relationship*
I don’t tend to go to the Drs for any treatment for it, cos they don’t seem to know much about backs…It’s a different sort of thing isn’t it the way the Dr treats you…. Uhm they just diagnose things don’t they… I find my Dr a very sympathetic Dr, so I can easily you know, if I have problems I tell him. He’s a really good Dr, he’s lovely… he’s really sort of enthusiastic. So he’s nice, he’s a good doctor.(P4)
She was very very good… very involved, and I felt that she knew me as a person, that when I was going I was actually getting really good one to one treatment… I felt she cared about what she was doing and that was really important to me. I felt I had a really good rapport with her. I felt that she had a sensitivity to how I was feeling and she gave me great consideration, and she empathised and awful lot with what I’d been through and the problems that I’d had. I built a very big sort of bond with her because I trusted her and what she was doing, and that to me was a big issue, that I actually trusted somebody to give me the treatment after having really problems before… that was really important (P9).
But I think with backs GPs just don’t know what they are talking about. They just don’t know. You have to go to someone that is a specialist…. if I do need to go back then I shan’t go to my GP. When I went I didn’t actually see my GP, I saw a locum and she laid me on the bed and out me through some exercises, my leg this way, that way, examine my back and she said I really can’t l do anything for you… She gave me a couple of sheets to do some exercises. I only did them for one day, it nearly killed me. I was in total agony, so I thought this is a waste of time (P10)
I don’t think it is doing me any good you know and yet I keep going and paying them money.. their care of you and their courtesy is you know second to none, I mean you just can’t fault it, its absolutely superb you know its absolutely superb…. they are so nice, and their care of you and their concern for you and the privacy and everything. That’s very special, and you don’t get that from your GP do you? They are so patient with you, so kind and so non-judgemental. (P11)
*Lifestyle advice*
I’ve got a good strong back, I could lift you up; like that but it wouldn’t do my back any good. You can actually do it, you can use all you muscles, physically and sometimes it’ll be perfectly alright and other times all you have to do is go like that and it goes you know. (P3)
She asked me once if I felt the weight had anything to do with it… I can remember we talked about something to do with that,…they only tell you to lose weight if you go to the Dr, and I said I’ve tried and tried over years and years losing weight and I didn’t want to be told again, you know. So it wasn’t like an option to help me… The things is, things are easing, but it’s nothing to do with the acupuncture, it’s because I’m losing some weight (P4)
I do my yoga, I can’t do the full lotus, I can’t do the splits yet, I’m not happy with my neck because I can’t do the plough. I can’t switch over and put my knees there. I’m not happy. Yesterday I got off the floor in a full crab… going backwards is hard tilting backwards is painful, it doesn’t hurt until I try to bend backwards to do a full crab. I’m doing my exercises every morning… I’ve been to the gym and there are special exercises I can do to build up my back.(P6)
We looked at diet which is quite important… At foods that were cold, foods that were hot, foods that would benefit me because my body had gone cold with the anaesthetic. (P9)
Don’t do anything you shouldn’t do. Don’t go lifting heavy weights… Just take it easy, don’t do things… She said no I don’t want you to go to the gym… take it easy… she did give good advice, which all except once when I was doing the gardening I followed. I wouldn’t say it learned me an awful lot.(sic) (P10)
She said I should try some yoga exercises and she showed me a couple of exercises how to stretch from my ankle right up through my hip and how to stretch over to one side and then you turn over and do the other side…the recommendations were yoga exercise and that and to go to yoga generally, that would help me relax.(P12)
Patients involvement in recovery
Like any acupuncture session, there was a good question and answer session beforehand and she summed up well saying to me you eat well, walk well, act well, and here is nothing really to change in your lifestyle. I am certainly overweight with lack of walking but that will come down this year (P1)
I stand over this table which is probably not high enough for doing the job. I can tell when I’ve been doing a job like that because later on in the day I get back pain.(P4)
I have to take responsibility for getting into this mess. It’s a hard lesson isn’t it, We are in charge of what we do. I will have to make certain changes. I’ve got to get fitter, I’ve got to be more disciplined in doing exercise to strengthen my back and this whole area so it won’t happen again, I have to really think about my posture 100% of the time, when I am standing, when I’m sitting …. Be aware and perhaps things then become second nature. (P5)
It makes you take control of your life because you have to make a decision to go to acupuncture in the first place, so you’ve got to a point where you need to do something… It’s like a first step, now I’ve sorted my back out, now I can start living.(P6)

## Results

### Participants

The patients interviewed were 2 men and 10 women, purposively sampled to reflect the diversity of known characteristics across all participants in the trial. In the three-month questionnaires, which were completed at around the same time of the interviews, all study participants reported their health as fair to excellent, and the majority reported satisfaction with the acupuncture they had received. Only half of the patients who were interviewed reported satisfaction with the level of their back pain at three months.

### Themes

Three over-arching themes pertaining to the acceptability of acupuncture treatment were identified. The first over-arching theme entitled “Facilitators of acceptability” contained five subthemes; reduction in symptoms, improved physical activity, relaxation, reduction of psychological symptoms and reduced reliance on medication (Table1). The second over-arching theme entitled “Barriers to acceptability” encompassed four subthemes: needle-related discomfort, temporary worsening of symptoms, and tiredness, and the pressure to continue treatment with its’ potential financial cost (Table2). The third over-arching theme entitled “Mediating factors” comprised pre-treatment mediators (Table 3), and treatment related mediators with four separate subthemes; time, therapeutic alliance, lifestyle advice, and patient’s active involvement, each with the potential to induce positive or negative influences on the patients’ perception of the treatment.

### Facilitators of Acceptability

#### Reduction in symptoms

The first facilitator of acceptability of treatment to be identified from the coding was the subtheme “reduction in symptoms of low back pain"(Table1). Two patients reported experiencing a reduction in pain symptoms from the first treatment session. More commonly, the pain relief became apparent gradually, following a trajectory over a period of several weeks. For example, several patients reported that they noticed a reduction in symptoms of low back pain about 4–6 weeks into treatment.

*“I had gone at first where it ached for two or three sessions, and I thought, this is not going to work, and then on the fourth session it was a lot easier. It was either the fifth or the sixth session when it was severe, and after that great. Each time I went then I got better and better. It got to the last one and I thought well really I don’t need it”.* (p10)

Through the patients’ descriptions of pain relief, different types of pain were identified; commonly, severe pain was referred to in threatening terms such as sharp, stabbing, burning, whilst dull and aching pain was considered more tolerable. Two patients reported a reduction in their severe pain, to leave a dull aching, which they considered a good outcome. The relief from constant pain was reported in some cases, whereas for others, the specific types of pain changed in nature. Several patients reported a reduction in the frequency of radiating nerve pain or sciatic pain, bodily dissociation and numbness associated with back pain. One patient graphically described improvement as a sensation of energy flowing, warmth generated, and “*the nerves have come back to life*” (p7). Most patients still experienced a degree of pain during specific household or work related tasks.

*“I don’t have constant pain like I did before, I know when I do certain things I'm going to get pain, but I don’t have it constantly which is a big difference to me in my life”* (p12)

The reduction in painful symptoms was not limited to low back pain; two patients reported that their chronic knee pain had been relieved, and another patient was pleased that her shoulder pain was treated during the course of treatment. In summary, the perceived reduction in the symptoms of back pain appeared to facilitate the acceptability of acupuncture.

#### Improved physical activity

A second subtheme to facilitate acceptability of treatment was the improvement in energy levels and physical functioning. For the majority of patients the awareness of the reduction in daily pain became apparent through their ability to increase physical activity. Several patients compared their levels of activity before and after acupuncture. Their descriptions allude to the debilitating nature of chronic pain and illustrate how pain regularly interrupted everyday tasks, whereby short periods of rest were necessary in order to complete the task in hand. Over the course of treatment, several patients noticed an increase in their level of energy, which enabled them to complete daily chores more easily.

*“My energy level has increased. I can now cook a meal without having to lie down every couple of minutes in between doing something, which is better”.* (p5)

The salience of improved physical function differed; for one patient the ability to conduct self-care and simple activities of daily living were a very positive aspect. For several others the improvement in energy levels meant they could engage in beneficial exercise, an aspect that helped to focus the patient on their abilities rather than disability. This served to reduce frustration and, for some, led to being able to engage in gentle exercise activities to help regain strength and fitness. These positive outcomes in functioning were a welcome effect of the treatment.

*“You can get about; you can do things you’re not just kind of stuck to a chair or confined to the house… I like to go swimming which you know I couldn’t do and I’ve done it since the treatment. I’ve been back swimming I do that, and I hoover up now, which I didn’t do before and I’ve done a lot of walking”* (p8).

#### Relaxation

A third facilitator of acceptability of treatment was a positive, and for some unexpected, side effect of treatment, namely relaxation. Within this subtheme, several patients reported feeling very relaxed whilst the acupuncture needles were in situ, although the interpretation of relaxation differed between individuals.

*“While I was there having the treatment I felt relaxed…it was just nice to be peace and quiet and just lay there… It’s a relaxant, it just levels you, it levels you an talking relaxes you so that you’ve got time…you walk home floating”* (p6)

One patient cited a feeling of well-being, another reported being able to clear their mind during treatment. Two others actually fell asleep during treatment, with one person reporting feeling revitalised afterwards, an effect that continued into the following day.

In summary, independent of whether there was a reduction in symptoms of pain, the feeling of wellbeing and relaxation during the treatment enhanced acceptability of the treatment.

#### Psychological symptoms reduced

The fourth subtheme of facilitators of acceptability was the psychological change experienced by some patients. Three patients linked the psychological components and physical components of chronic pain from different perspectives. One patient was clear in saying that the “*awful*” pain experienced from neural symptoms was the cause of their low mood and tearful state (p11). Another acknowledged that stress was a major contributor to poor posture and other physical problems.

*“You get a load of stress in your head and your body doesn’t work properly, you know that stress is transmitted into your posture and everything else”.* (p3)

This patient explicitly acknowledged the benefit of a holistic approach, where the acupuncturist was able to prioritise the treatment of his psychological symptoms. Unfortunately, for one patient the lack of ability to find suitable work due to chronic pain and the worry of the subsequent financial difficulty created extreme anxiety. Despite these worries the patient said, “*I’ve been listened to… I felt supported”*. (p5) The ability to share the emotional or psychological aspects of chronic pain with a supportive practitioner, who could include these aspects in the treatment, was an important element of acceptability.

#### Reduced medication

The fifth subtheme identified as a facilitator of acceptability was the reduced use of medication. Several patients reported reliance on a combination of over the counter anti-inflammatory medication and prescribed analgesics. One patient recounted how he took analgesics as a prophylactic measure before engaging in exercise. Another used analgesics as an aid to sleep.

*“Prior to the acupuncture I was taking pain killers for my back to get to sleep at night, and then as the acupuncture progressed I didn’t take any for my back… I don’t like taking pills because of their effects on the body.”* (p1)

Most patients disliked being dependent on medication, citing unpleasant side effects, fear of addiction and ineffectiveness of commonly used analgesics as major drawbacks. Once acupuncture treatment had started, one patient stopped taking medication as a means of testing the efficacy of acupuncture. Several patients reported a reduction in the dosage and frequency of usage over the course of the treatment. Overall, reduced reliance on medication, along with reduced concern about unwanted side effects was a further contributor to the acceptability of acupuncture.

### Barriers to Acceptability

The over-arching theme of barriers to acceptability comprised three subthemes related to treatment - needle-related discomfort, temporary worsening of symptoms, and tiredness - and a further subtheme on the pressure to continue with treatment with its potential financial cost (Table 2).

#### Needle related discomfort

The first barrier was associated with needling discomfort. Discomfort due to needling varied across several patients; in most cases the type of treatment reactions reported were transient and mild.

*“Sometimes the needles themselves, when they put them in a certain point, they twist them and you get a sharp pain, and they do it again till it’s a kind of achy pain”* (p5)

One patient described a possible needling injury; one of her fingers twitched for four days after treatment and she/he was concerned that a needle had hit a nerve (p6).

Another patient reported finding the treatment somewhat painful at times, but acknowledged that subsequent physical activity may have contributed to their discomfort.

#### Reactions to treatment: Temporary worsening of symptoms

Two patients reported an unpleasant worsening of back pain symptoms after treatment, which lasted into the following day, and was relieved by taking paracetomol. One patient had been warned of the possibility of a reaction. The warning helped to reduce the patient’s concern and enabled them to view the reaction as an acceptable temporary discomfort with potential for overall improvement (p11). In contrast, warning of a possible reaction led the other patient to assume that the discomfort they experienced was entirely due to the treatment. They did not link the worsening of their symptoms to the effects of an extended car journey taken immediately after treatment.

*“I got the most awful back-ache in the car…really really bad backache. I ended up taking paracetomol that night. I think it was a temporary thing, a reaction to the acupuncture ‘cos in fact she warned me I may get an adverse effect to it to start with and of course I did”,* (p4)

#### Reactions to treatment: Tiredness

Three patients felt extremely tired during the earlier sessions of the course of treatment. For one this was quite unexpected and inconvenient when lasting into the following day. In contrast, the second patient found the tiredness difficult to cope with initially, but later construed this reaction as an acceptable part of the healing process. The third patient successfully managed the situation by carefully planning appointment times. In doing so, the tiredness they experienced became part of their relaxation routine, and was viewed as recovery time.

*“I used to feel very tired… It was just a progression maybe of just your body saying you know you needed time to heal…I intentionally used to make my appointments later on in the day so I could come home and relax.” *(p9)

Several patients had previous experience of acupuncture and expected minor discomfort as an acceptable risk as part of the treatment. Although some patients continued to experience unpleasant reactions in later sessions, they had also experienced some benefit and persevered with the course of treatment. For a minority, these reactions detracted from the overall acceptability of the treatment, particularly where any overall benefit from treatment was less than expected.

#### Pressure to continue treatment and potential financial cost

The fourth barrier to acceptability was the pressure to continue with treatment after the course of treatment paid for by the YACBAC trial had ended. None of the twelve people interviewed reported 100% pain relief without later recurrence of some of the back pain symptoms. Several patients were still in pain at the end of the 10 session course and reported that their acupuncturist had suggested one or two (or more) treatments as a private patient. Two patients welcomed the opportunity to continue. Another, who had not experienced pain relief felt unsure, but had appreciated the care and concern they had received and agreed to pay for additional sessions in the hope that it would work eventually.

*“She suggested it; it was her who suggested five more because she thought… she was hoping we would have cracked it really in five more sessions. I mean that must have been at the time when we were still feeling there was some improvement being maintained”.* (p12)

Two more patients would have liked to continued treatment, or would have considered treatment in the future, but were constrained by the financial cost of each session. Another patient tried to have the treatment continued via the GP but was told that funds were not available. In summary, patients found the cost of treatment more acceptable if they were experiencing some benefits, however, where the benefit was in question, or if they felt pressured to pay or could not pay, then the pressure to continue treatment and the potential financial cost of ongoing treatment were not acceptable.

### Mediating Factors

The third over-arching theme is that of mediating factors, which could potentially influence a patient’s behaviour, experience and perception of treatment in either positive or negative ways. Pre-treatment mediating factors related to aspects of expectation and previous experience (Table 3). Two treatments related mediating factors, that of time and the therapeutic alliance highlighted contrasts between the consultations with their acupuncturist and their General Practitioner. Two further treatments related mediating factors were life style advice designed to promote and sustain a reduction in back pain and the patient’s active involvement, both of which were influenced by the type of advice offered to patients, and the willingness to act on the advice.

### Pre-treatment Mediators

#### Expectation and experience

Expectation of efficacy varied; six patients with previous experience of acupuncture felt that it might be helpful. For those without previous experience, two patients expressed doubt, and were influenced by the attitudes and experiences of others, one had read extensively and was very positive. Another felt excited at the prospect, but realised that the effect may be limited; one was convinced that belief was the key to efficacy. Most patients welcomed the opportunity to try acupuncture because their pain was severe, and they disliked taking medication. Only one patient, based on previous experience, offered an explanation of how he expected acupuncture to work:

*“with acupuncture it tends to keep the energy flowing through everything so it can obviously work better…if you’ve got energy going through properly which is what acupuncture does really, it controls these hidden energy lines which goes through and activates or deactivates muscles really or invigours(sic) them, so if you can organise that and get that kind of thing balanced then your back’s going to be straight and that’s how I think it works for me anyway”* (p3).

### Treatment Related Mediators

#### Time

Time, the first mediating subtheme, represented a component of treatment that had a multifaceted impact that underpinned all aspects of the treatment process. The majority of patients interviewed enjoyed the time spent within an acupuncture consultation and contrasted it with that of a GP consultation. The acupuncture consultation was found to be more acceptable by several patients because it allowed them time to fully explain their experience of pain, receive individualised treatment for their back pain and other concurrent physical and psychological symptoms within the same consultation.

*“It’s totally for you, not for anybody else, totally for you and its lovely to have the attention instead of being on this five minutes list at a doctors and they don’t listen. They’ve got to listen to you and she’s interested”.* (p6)

One patient reported her enjoyment of the acupuncture session as a personal time to relax and reflect. In contrast, another patient who had a pressing workload found the treatment too time consuming for the pain relief gained and resented setting time aside to attend sessions. Even where GPs had given a diagnosis, prescribed several medications and made a referral to a physiotherapist, the brevity of the GP consultation was the overriding memory for most patients. For most people interviewed, the disparity in the time allowed for the GP consultation enhanced the perceived acceptability of the acupuncturists’ consultation.

*It’s a very nice sort of atmosphere there all friendly there and very relaxed. It’s not like sitting in the doctor’s waiting room you know pretending not to look at each other and waiting… You know it’s completely different … well it’s holistic compared to hustle-istic in the doctors.* (p3)

#### The therapeutic alliance

Underpinned by the subtheme of time, the second mediating factor was the subtheme of the therapeutic alliance. On a superficial level, three patients reported that their acupuncturist was kind, considerate and friendly. For two patients the development of good rapport and a trusting bond were key components of the therapeutic alliance and instilled faith in the acupuncturist’s abilities.

*“She’s well trained to do things, she’d know what she was doing… they are more thorough in examining you and ironing out your problems”* (p5)

For one patient, the level of courtesy and privacy was a special feature of the acupuncturist’s care. Two patients also comment on the acupuncturist’s care, concern and understanding of their condition compared to a perceived lack of sympathy from their doctor. Although one patient considered their doctor enthusiastic and sympathetic, the patient still lacked confidence in the doctor’s knowledge of treatment for back pain.

*“I think, with backs, GPs just don’t know what they are talking about. They just don’t know. You have to go to someone that is a specialist… if I do need to go back then I shan’t go to my GP”* (p10)

In contrast, where an acupuncturist was working in a multi-bed setting in a college clinic and moving between several patients at a time, the attention was not as personal, and the patient was left to glean information from passing acupuncture students.

In summary, for most patients the provision of sufficient time and one-to-one attention clearly facilitated the development of the therapeutic relationship and contributed to the acceptability of the treatment.

#### Lifestyle advice

The third component within the theme of mediating factors was the provision of appropriate lifestyle advice. For the majority of patients, lifestyle advice was a common feature in consultations within both acupuncture and GP care. Patients were offered lifestyle advice related to their acupuncture diagnosis over the course of treatment. For several patients, the supportive encouragement from the acupuncturist helped them to engage in gentle exercise and regular activity according to their individual ability and with realistic expectations. In contrast, one patient seemed to be irritated with advice on exercise as at the time they regularly engaged in exercise that they considered appropriate to help them regain function and mobility. Three patients received dietary advice to lose weight, whereas another was offered individualised dietary advice to help them understand the importance of warm and cold foods in relation to their traditional Chinese diagnosis. Several patients received advice from the acupuncturist about their posture when walking:

*“She did give me two exercises to do with my hips which I have tried to do. She also talked to me about trying to look at myself in the mirror and see, try to be aware of my posture and also try to walk… keep my hips mobile and keep them moving in the right way really. So it’s been more to do with physical posture and things like that”* (p11)

Two patients were advised to take more rest. One of these was specifically asked to do exercise to strengthen their back muscles but very clearly not to go beyond the point where the pain would get worse. The second was advised not to do anything, and to take it easy, and the patient summed up that “I wouldn’t say they *learned me an awful lot”* (p10).

Overall, lifestyle advice was reported to be more acceptable when the acupuncturist provided it gradually, over the course of treatment**.**

#### Patients’ active involvement

The fourth mediating factor was the subtheme of the patients’ personal involvement in their own recovery. Three people reported that the treatment had brought them to an acceptance of their back pain. Two of those accepted that there was always going to be a certain amount of pain, but they could live with that. Another two patients became more aware of their fragility, but managed their back pain by maintaining personal vigilance of their posture and engaging in specific strengthening exercises.

Two patients reported regaining a strong sense of control of their life and cited taking personal control as a key feature of their ongoing recovery, and the importance of setting time aside for themselves. Although acupuncture was not particularly effective for another person, they felt better placed to seek other treatment rather than putting up with back pain. In contrast, two people knew what steps to take in order to manage their back pain, but were unwilling to make changes. One other patient felt that he had taken all lifestyle steps possible to ensure a good recovery, and though capable of lifting heavy objects, he remained fearful of taking a labouring job that might lead to re-injury. In summary, for most patients, the taking of responsibility and being supported to gain control were potential facilitators of acceptability.

*“The start of curing yourself is to take steps to do something about it. That’s an acceptance, you know something’s wrong with you and you’ve got to get it sorted out. It does help to create a positive attitude. I deliberately altered me posture, trying to get it back over a year or two”.* (p3)

## Discussion

### Key Findings

The results of this study show that acceptability of acupuncture for patients is based on a complex and multifaceted appraisal of the treatment, which incorporates potentially positive and negative experiences and perceptions. The therapeutic relationship between the practitioner and patient appears to be a strong driver of acceptability, but not the only reason. In addition to offering care and compassion, the supportive therapeutic alliance is a useful vehicle to promote learning, and to develop the patient’s self-efficacy in pain management, two aspects that might contribute towards a beneficial outcome and its maintenance. In this study, patients reported a range of beneficial outcomes and drawbacks of treatment. However, the judgment on how each of the benefits and drawbacks contributed to acceptability varied with the patients’ individual experience. These qualitative findings should be considered in the context that the quantitative data showed acupuncture had a clinically and statistically significant effect in reducing chronic pain. An understanding of the underlying mechanisms of effectiveness was not considered important by patients in their reports on efficacy or acceptability of treatment.

### Strengths and Limitations

Qualitative studies nested within randomised controlled trials are particularly suited to exploring in depth the reasons why an intervention might be successful or not. Our use of in-depth interviews provided a deep understanding of what acceptability means to patients receiving acupuncture for low back pain and presented rich detail of how they interpret their experience of treatment. The bottom-up process of thematic analysis was conducted following the steps recommended by Braun and Clarke. [15] This method allowed the themes to develop directly from the patients’ own voices, and enabled a more accurate representation of their experiences without the constraint of preconceived ideas.

Our findings provide insight into a key factor related to effectiveness, namely acceptability of treatment. Acceptability is an important factor because it will impact on the take-up of the treatment offered and the compliance or willingness to see the course of treatment through. We have reported on patients’ experiences of acceptability, whether the outcomes from acupuncture were beneficial or not. A strength of our study is that we have been able to qualify the quantitative results on effectiveness with our data on acceptability. A better understanding of the underlying factors that impact on acceptability and effectiveness enhances our understanding of the potential transferability of the intervention to practitioners of acupuncture across the UK. For example, our study adds to the quantitative data by providing an understanding on the impact of the patient-practitioner relationship on the acceptability of acupuncture treatment, and what steps the practitioner and patient might take to maximise the likelihood of acceptability for the patient.

A limitation of the study was the small number of interviews, however, the coding for each theme was congruent across the majority of the sample and data saturation occurred within six interviews. Individual differences were included in the results to illustrate contrasts where they occurred, and to avoid bias in representation. The researchers AK and HM were in accordance with each other on the data extracted and their interpretation; however lack of wider consultation may be a potential source of bias. A further limitation was the minimal integration with the quantitative data available from the YACBAC trial. [16] Although the qualitative data presented here support the quantitative findings that the majority of patients were satisfied with the treatment received in terms of time and attention and were willing to try acupuncture again, the quantitative data provided insufficient depth of detail to crosscheck and integrate with the components of acceptability reported in this study.

### Comparison with Other Studies

Willingness to have acupuncture again has been found to be a quantitative indicator of acceptability among patients with low back pain. [16] Our previous paper on the willingness to have acupuncture after experiencing a reaction to treatment reported that the benefit of reduced back pain over the course of treatment outweighed negative experiences associated with treatment reactions. [17] The qualitative analysis conducted in the current study is consistent with these previous findings and complements them by identifying the therapeutic alliance and the active engagement of the patient in their own recovery as a driving force for beneficial change.

The expectation of pain relief is reported to have a significant impact on outcomes in patients with chronic pain. [18]; [19] However, in this study, although expectation was a pre-treatment mediator of acceptability of treatment, the patients with no experience of acupuncture had a higher expectation of pain relief and reported less benefit than anticipated. In contrast, those who had previous experience of acupuncture were more realistic in their expectation of efficacy. This finding is consistent with the quantitative data from the YACBAC trial population that reported weak evidence of an interaction effect whereby positive belief of those receiving acupuncture was associated with less benefit [16]. The finding is also consistent with research which suggests that perceived outcomes are related to patient-practitioner relationship factors [20]; [21].

An awareness of gaining greater control over back pain enhanced the acceptability of the treatment for some patients. Within the therapeutic alliance, the development of the learning processes appeared to engender greater efficacy in the patients’ personal management of back pain. These findings are consistent with practitioner reports from two other qualitative studies[22]; [23]. In contrast, acceptability diminished in patients who reported a reluctance or inability to put self-care into practice or make adaptive changes to working practices. This was more apparent where patients continued to expect the acupuncturist to provide an immediate and long lasting cure, and supports Foster’s [24] finding that the lack of belief in a personal ability to manage back pain and the assumption of the inevitability of a future with pain, are major psychological obstacles to recovery.

Acceptability of acupuncture treatment appears to increase when the combined processes of care lead the patient to accept responsibility for their ongoing back care, and helps to shift their focus from pain elimination to the restoration of function. This shift in focus creates of an opening for a transition from the biomedical model which focuses on healing through techniques, toward the ‘healing through adaptation,’ based on a biopsychosocial explanatory model of back pain [7].

### Implications for Practice and Future Research

The time allowed for each acupuncture session was an important contributor to acceptability, however, the time-frame needed for the processes of treatment to occur is not possible within the consultation time available within the GP surgery. [6] The partnership developed between the therapist and patient was a key factor of acceptability, this opened up the potential for learning processes to be utilised as a means of increasing patient’s self-efficacy in pain management. Our research has reinforced the value of improving patient’s perceptions of their personal control over pain and of reducing the expectation of the inevitability of an ongoing back problem and passive progression to disability. Looking at acceptability in this way has opened up the possibility of investigating the mechanisms of action from the patients’ perspective, and generates the hypothesis that increased self-efficacy is one such mechanism that may underpin the long-term effects of acupuncture. These qualitative data suggest that the inclusion of a self-efficacy measure [25] in a randomised controlled trial could identify whether those patients who may be already predisposed to improve irrespective of treatment do better, and determine whether or not other patients can gain efficacy in pain management through the processes of acupuncture treatment. Secondly, the optimum time after the onset of back pain for acupuncture treatment to reduce pain and prevent chronicity and disability is unknown.

### Conclusions

Acceptability of acupuncture treatment for low back pain is associated with a complex appraisal of the treatment processes and outcomes, and reflects the quality of the care received. Acceptability is enhanced by beneficial physiological and psychological outcomes, and the development of learning processes that engender personal responsibility and increased self-efficacy in the management of low back pain. Dissatisfaction with the amount of pain relief received, and unpleasant treatment effects do not necessarily detract from the patients’ overall perception of acceptability of the treatment.

## Supporting Information

Appendix S1 **TOPIC GUIDE.**
(DOC)

Appendix S2 **COREQ 32-ITEM CHECKLIST.**
(DOC)
