# Supplementary material for: The Acceptability of Acupuncture for Low Back Pain: A Qualitative Study of Patient’s Experiences Nested within a Randomised Controlled Trial
Source: PLoS One. 2013 Feb 21;8(2):e56806. doi: 10.1371/journal.pone.0056806 (PMC3578863; doi:10.1371/journal.pone.0056806)
Supplement: Appendix S1 — TOPIC GUIDE. (DOC) [file pone.0056806.s001.doc]

APPENDIX S1: TOPIC GUIDE

INTRODUCTION:

What I’d like to talk with you about are you experiences and views of acupuncture as a treatment for back pain.

BACK PAIN

Before we discuss your experience of acupuncture, could you tell me the brief history of you back pain from when it firs started up to the point you began having acupuncture?

What have you tried over the years to make your back pain better?

ACUPUNCTURE EXPERIENCE

I’d like to move on now to you actual experience of acupuncture itself

- Expectations
- Thoughts when GP told you about the study
- Had acupuncture before
- Think acupuncture could help
- Ideas about how acupuncture might work and what was involved
- Expectations of acupuncturist

Intervention

What has the treatment involved for you?

How many treatments did you have? Did you find it sufficient?

- Information and communication
- What did you acupuncturist tell you about the treatment?
- How did they explain things to you?

Outcomes

Have you noticed any changes (physical) as a result of the treatment?

- Improvement in back?
- Increase in activities?
- Reduce /stop medication?

Apart from physical changes have you felt any better in yourself as a result of the acupuncture (emotional changes)

Do you think having acupuncture might have changed the way you see your back problem?

How do you feel about your back problem now compared to before you had the acupuncture treatment? Did you get what you were hoping for out of the acupuncture?

Experiences

What is it like going for acupuncture?

How did you feel when you were having acupuncture (relaxed, energised, adverse reactions?)

Have you been happy with the treatment you received from you acupuncturist? Is there anything you haven’t liked or that has worried you?

Therapeutic relationship

Could you tell me about the relationship you had with you acupuncturist?

Was the relationship with your acupuncturist similar or different to the relationship you have with your doctor?

ACCEPTABILITY OF ACUPUNCTURE

Would you recommend acupuncture to other people with back pain?

Would you try acupuncture again for a health problem?

What reasons do you think other people may have for not wanting acupuncture?

Is there anything else you would like to tell me or you think I should know?
